# Inhibitive Effects of FGF2/FGFR1 Pathway on Astrocyte-Mediated Inflammation *in vivo* and *in vitro* After Infrasound Exposure

**DOI:** 10.3389/fnins.2018.00582

**Published:** 2018-08-24

**Authors:** Ya-Jun Shi, Ming Shi, Li-Jun Xiao, Li Li, Lin-Hui Zou, Chao-Yang Li, Qin-Jun Zhang, Lin-Fu Zhou, Xin-Chao Ji, Huan Huang, Ye Xi, Ling Liu, Hong-Ya Zhang, Gang Zhao, Lei Ma

**Affiliations:** ^1^Department of Neurology, Xijing Hospital, The Fourth Military Medical University, Xi’an, China; ^2^31668 Troops of PLA, Army Medical University, Xining, China; ^3^Department of Psychological Medicine, The General Hospital of PLA, Beijing, China; ^4^Department of Neurology, Meishan Cardio-Cerebrovascular Disease Hospital, Meishan, China; ^5^Department of Neurology, Third Hospital of PLA, Baoji, China; ^6^Department of Neurobiology, School of Basic Medicine, The Fourth Military Medical University, Xi’an, China

**Keywords:** infrasound, hippocampus, astrocytes, FGF2, FGFR1, NF-κB, neuroinflammation

## Abstract

Infrasound, a kind of ambient noise, can cause severe disorders to various human organs, specially to central nervous system (CNS). Our previous studies have shown that infrasound-induced CNS injury was closely related with astrocytes activation and astrocytes-mediated neuroinflammation, but the underlying molecular mechanisms are still largely unclear. FGF2/FGFR1 (Fibroblast growth factor 2/Fibroblast growth factor receptor 1) pathway was reported to play an important role in anti-inflammation in CNS disorders. To further study the possible roles of FGF2/FGFR1 pathway in infrasound-induced CNS injury, here we exposed Sprague-Dawley rats or cultured astrocytes to 16 Hz, 150 dB infrasound, and explored the effects of FGF2 on infrasound-induced astrocytes activation and neuroinflammation. Western blotting, immunofluorescence and liquid chip method were used in this experiment. Our results showed that after 3- or 7-day exposure (2 h/day) of rats as well as 2 h exposure of cultured astrocytes to 16 Hz, 150 dB infrasound, astrocyte-expressed FGFR1 was downregulated *in vivo* and *in vitro*. FGF2 pretreatment not only inhibited infrasound-induced astrocyte activation in rat hippocampal CA1 region, but also reduced the levels of pro-inflammatory cytokines, such as TNF-α, IL-1β, IL-18, IL-6, and IFN-γ *in vitro* and *in vivo*. However, FGF2 significantly upregulated the expression of FGFR1. Furthermore, we showed that FGF2 could attenuate IκBα phosphorylation, NF-κB p65 translocation, pro-inflammatory cytokines levels, and neuronal loss in the CA1 region induced by infrasound. On the contrary, PD173074, a special antagonist of FGFR1, could reverse the effects above *in vitro* and *in vivo*. Taken together, our findings showed that FGF2/FGFR1 pathway may exert inhibitive effects on astrocyte-mediated neuroinflammation *in vitro* and *in vivo* after infrasound exposure.

## Introduction

Infrasound is a common public health problem produced by all kinds of noise in the environment (Leventhall, 2007). Infrasound is also a sound of low frequency between 0.0001 and 20 Hz (Sand and Karlsen, 2000; Alves-Pereira and Castelo, 2007). Its essence is mechanical vibration. Infrasound comes from two aspects, natural world and artificial activities, including volcanic eruptions, tsunami, aircraft flight, and weapons (Angelis et al., 2016). Infrasound can cause a systemic disease, known as vibroacoustic disease (VAD) (Ferreira et al., 2006; Mendes et al., 2006; Alves-Pereira and Castelo, 2007), which may cause damages to many systems, such as the central nervous system (CNS) (Yuan et al., 2009; Cheng et al., 2012; Zhang et al., 2016), digestion system (Da et al., 2006), cardiovascular system (Pei et al., 2009), respiratory system (Castelo et al., 2003; Branco et al., 2007), and other systems.

Infrasound can cause VAD, though the specific mechanism is unknown. It has been suggested that infrasound can induce neuronal axon degeneration and neuronal damage by promoting Ca^2+^ influx (Cheng et al., 2012). Exposure to infrasound can remarkably impair the learning and memory ability of rats (Liu et al., 2010; Ma et al., 2015) and increase the neuronal apoptosis in rat hippocampus because of pro-inflammatory cytokines from neuroglial cells (Yuan et al., 2009; Shi et al., 2013; Cai et al., 2014). Under the infrasound with frequency 16 Hz and sound pressures ranging from 90 to 130 dB, the average escape latency of rats was increased, indicating that the more infrasound was intensified, the more learning and memory was damaged (Shi et al., 2013).

Our previous study revealed a relationship between infrasound-induced CNS injury and glia-triggered neuroinflammation (Shi et al., 2013). Glial cells were activated by infrasound exposure of 16 Hz, 130 dB and the levels of IL-1β and TNF-α in glial supernatants were increased, suggesting that anti-inflammation may be a new therapeutic approach to attenuate infrasound-mediated injury (Shi et al., 2013; Cai et al., 2014). However, we still have little knowledge about the mechanism underlying glial cell-mediated inflammation under infrasound exposure. Several lines of evidence revealed that FGF2/FGFR1 pathway played an important role in neuroinflammation as well as its effect on cell proliferation, differentiation, and neurogenesis (Yoshimura et al., 2001; Frinchi et al., 2010; Celik et al., 2016). For instance, exogenous FGF2 inhibited hippocampal inflammation induced by LPS and reversed the neuroinflammation-induced depressive-like behavior (Tang M.M. et al., 2017).

In this study, using infrasound-exposed rats and astrocyte culture, we respectively activated and blocked FGF2/FGFR1 pathway by FGF2 and FGFR1 antagonist PD173074 to study its effects on infrasound-induced inflammation. Our results revealed that infrasound exposure induced astrocyte activation and downregulated the expression of FGFR1. FGF2 administration could alleviate activated astrocyte and upregulate FGFR1 expression, and however, PD173074 reversed these effects.

In this study, to investigate whether FGF2/FGFR1 pathway attenuates astrocytes-mediated inflammation after infrasound exposure, we respectively activated and blocked FGF2/FGFR1 pathway by FGF2 and FGFR1 antagonist PD173074 to study its effects on infrasound-induced inflammation *in vivo* and *in vitro*. Given that astrocyte-mediated inflammation and anti-inflammation effects of FGF2, we proposed that infrasound may induced astrocyte-mediated inflammation by inhibiting FGF2/FGFR1 pathway and activating FGF2/FGFR1 pathway might be a promising way to treat infrasound-associated diseases.

## Materials and Methods

### Infrasound Generation

High-intensity infrasound chamber and detection system, called as the infrasound system, were built by the Fourth Military Medical University and Aerospace Industry Corporation, described in our previous studies (Du et al., 2010; Shi et al., 2013). The infrasonic pressure chamber system used in this experiment is composed of an air compressor, an air flow modulator, an infrasonic cabin and a set of software analysis system. The detection system consists of three parts: a infrasound sensor, a set of data acquisition system for ultra-low frequency-noise signal and a set of computer simulation analysis system. The infrasound system uses the key techniques of high infrasonic excitation sources and realizes the continuous adjustable frequency of high-intensity infrasonic signal. The infrasound system can generate standard infrasonic waves with a frequency range from 4 to 20 Hz and a sound pressure level from 150 to 160 dB. In this experiment, a frequency of 16 Hz and a pressure level of 150 dB was performed.

### Animals and Drug Administration

Male SD rats (clean grade) weighting 220–250 g were obtained from the Experimental Animal Center, Fourth Military Medical University. The animals were housed under controlled condition with 12-h light, 12 h dark. The rats were provided by normal administration of rodent diet and tap water. The protocol was approved by the Animal Ethics Committee of Fourth Military Medical University and Use Board. All invasive procedures were performed under anesthesia with pentobarbital sodium (40 mg/kg) and under physiological monitoring such as respiration and blood pressure, minimizing discomfort for rats.

*In vivo*, all rats were equally divided into the control group (the rats without infrasound exposure, *n* = 6), the infrasound (IS) group (those exposed to 16 Hz, 150 dB of infrasound for 1, 3, or 7 days, *n* = 6 each), the FGF2 groups (infrasound-exposed rats treated with FGF2, *n* = 6 each), the PD173074 groups (infrasound-exposed rats treated with PD173074, *n* = 6 each). For FGF2 administration, the rats were injected intraperitoneally (i.p.) with 0.1 mg/kg. For PD173074 administration, the rats were injected i.p. with 1.5 mg/kg. FGF2 was dissolved in saline (Graham and Richardson, 2009; Graham and Richardson, 2010). PD173074 was dissolved in saline containing 12.5% cremophor EL and 2.5% DMSO (Di Marco et al., 2014). The rats were received with four doses of either FGF2 or PD173074 before 24, 16, 8, and 2 h of infrasound exposure.

*In vitro*, cultured astrocytes were given with FGF2 (200 ng/ml) or PD173074 (2 μM) at 2 h before infrasound exposure, and then divided into the control group, the infrasound group, the FGF2 group, and PD group. The dosages of FGF2 and PD173074 used in the present study were based on the previous research and our pilot experiment (Williams et al., 2003; Weiss et al., 2010). The purified astrocytes were reseeded at a density of 3 × 10^4^ cells/cm^2^ onto glass coverslips in 96-well plates (for immunofluorescence) or 1 × 10^6^ cells/cm^2^ in 6-well plates (for western blot). For each group, at least three assays were repeated.

### Reagents and Antibodies

Rabbit anti-FGFR1 was purchased from Abcam (Cambridge, United Kingdom), mouse anti-GFAP was from Novus (Colorado Springs, CO, United States). HRP goat anti-mouse IgG(H+L), HRP goat anti-rabbit IgG(H+L) were from Jackson ImmunoResearch (West Grove, PA, United States). Hoechst 33342 was from GeneCopoeia (Rockville, MD, United States). SDS-PAGE Gel Kit, SDS-PAGE Loading buffer were from Beijing ComWin Biotech, Co., Ltd. (Beijing, China). Recombinant rat fibroblast growth factor 2 was from Novoprotein (Shanghai, China). PD173074 dissolved in DMSO was from Selleckchem (Houston, TX, United States). NE-PER Nuclear and Cytoplasmic Extraction Reagents was from Thermo (Waltham, MA, United States). Milliplex MAP Kit Rat Cytokine Magnetic Bead Panel, Milliplex MAP Lysis buffer were from EMD Millipore (Millipore, Germany). DMSO was from Sigma (St. Louis, MO, United States). DMEM with glucose and L-glutamine was from Corning (New York, NY, United States). All the antibodies were placed at −20°C after dispensing, and stored at +4°C after used, except that the antibodies containing 50% glycerol were always placed at −20°C. All the antibodies were avoided freezing-thawing cycle.

### Preparation of Brain Tissues

At the end of infrasound exposure, rats were anesthetized as described above. The protocol for obtaining brain slices was based on previously described (Melvin and Sutherland, 2010). Then rats were intracardially perfused with 150 ml normal saline at 30 r/min, 200 ml 4% paraformaldehyde (PFA) at 30 r/min and finally 200 ml 4% PFA at 4 r/min by a perfusion pump. After perfusion, brains were placed in 4% PFA, fixed for 6 h and replaced with 30% sucrose solution, until the brain was at the bottom of bottle. Subsequently brains were frozen and sectioned in 30 μm thick slices at −20°C by a Leica CM 1900 cryostat. The brains slices were placed in a 12-well plate filled with 1× PBS and transferred to a 24-well plate containing 70% glycerol and stored at −20°C. These brains slices were used for immunofluorescence.

### Immunofluorescence

Double-labeling immunofluorescence was used to evaluate the co-expression of FGFR1/GFAP, cleaved caspase-3/NeuN and cleaved caspase-3/GFAP, as previously described (Kigerl et al., 2009). Cells and brain slices were washed with 1× PBS for 30 min, then blocked with 3% BSA for 30 min. The sections were incubated overnight at 4C in the following primary antibodies: rabbit polyclonal FGFR1 (1:200), mouse monoclonal GFAP (1:100), mouse monoclonal NeuN (1:800), Cleaved caspase-3 (1:200). After the sections were washed, primary antibodies were detected with dylight 488-conjugated goat anti-rabbit IgG or dylight 594-conjugated goat anti-mouse IgG secondary antibodies. Confocal images were obtained by using a Olympus FV1000 confocal microscope.

### Primary Astrocytes Culture

Primary cultures of neonate rat hippocampus astrocytes were prepared from 1-day-old rats, as previously described (Li et al., 2006; Hansson et al., 2008). Briefly, the hippocampus tissue of rats was collected in small dishes containing D-hanks solution and blood vessels were carefully removed. The tissues were cut by ophthalmic scissors and then 2 ml 0.05% trypsin was added. After digestion for 3–5 min, 4 ml culture medium was added to stop digestion and the cell suspension was filtered. Then the filtered cell suspension was centrifuged at 1000 rpm for 5 min at 4°C, and the cell suspension was cultured in DMEM supplemented with 10% fetal bovine serum, 1% glutamine, 1% penicillin and streptomycin under a 37°C incubator. If the culture medium turned yellow, it should be replaced with new medium.

### Western Blotting

Western blotting was done as earlier described (Block et al., 2013). Cytoplasmic protein and nucleoprotein was extracted from the primary culture cells in every group. According to the experimental procedure, equal amounts of proteins were loaded on 10% SDS-PAGE gels and transferred to membranes. Then the PVDF membranes were blocked with 5% skim milk for 2 h at room temperature and were incubated with rabbit polyclonal FGFR1 (1:400), rabbit polyclonal NF-κB p65 (1:500), rabbit monoclonal IκBα (1:1000), rabbit monoclonal p-IκBα (1:1000) overnight at 4°C. TBP (1:1000) and β-actin (1:2000) were used as an internal control. Corresponding second antibodies of HRP goat anti-rabbit IgG(H+L) and HRP goat anti-mouse IgG(H+L) were incubated for 1 h at room temperature. The enhanced chemiluminescence (ECL) system was used for detection to visualize proteins.

### Proinflammatory Cytokine Detection

Milliplex MAP Rat Cytokine Kit was used to measure the following pro-inflammatory cytokines: TNF-α, IL-1β, IL-18, IL-6, and IFN-γ, according to manufacturer’s instructions and as previously described (Arrode-Bruses and Bruses, 2012; Codices et al., 2012). All buffers and diluents were warmed to room temperature when used. The standard, quality controls, sample, buffer and beads were sequentially added in a 96-well filter plate and then were incubated for 2 h on the shaker at room temperature. For the detection of proinflammatory cytokine, the samples were respectively incubated with detection antibodies for 1 h and streptavidin–phycoerythrin for 30 min in the dark. After washing the beads twice with BioTek plate washer, the Median Fluorescence Intensity values were analyzed in the Luminex 200TM instrument by using the MILLIPLEX^®^ Analyst 5.1 analysis software.

### Data Analysis

All the data were expressed as means ± standard deviation (SD) and statistical analysis were performed using SPSS 16.0 software. Differences between groups were analyzed with one-way ANOVA followed by Tukey test. Each of the analyses groups met the normal distribution before ANOVA was performed. A value of *p* < 0.05 was considered statistically significant.

## Results

### Infrasound Downregulated the Expression of FGFR1

To examine whether FGF2/FGFR1 pathway is involved in infrasound-induced inflammation in astrocytes, we first observed the changes in FGFR1 expression after infrasound exposure. We found that FGFR1 and GFAP showed a co-localized expression pattern, and FGFR1 immunoreactivity appeared granular and was distributed on the surface of GFAP^+^ astrocyte membrane (**Supplementary Figure S1**).

**FIGURE 1:**
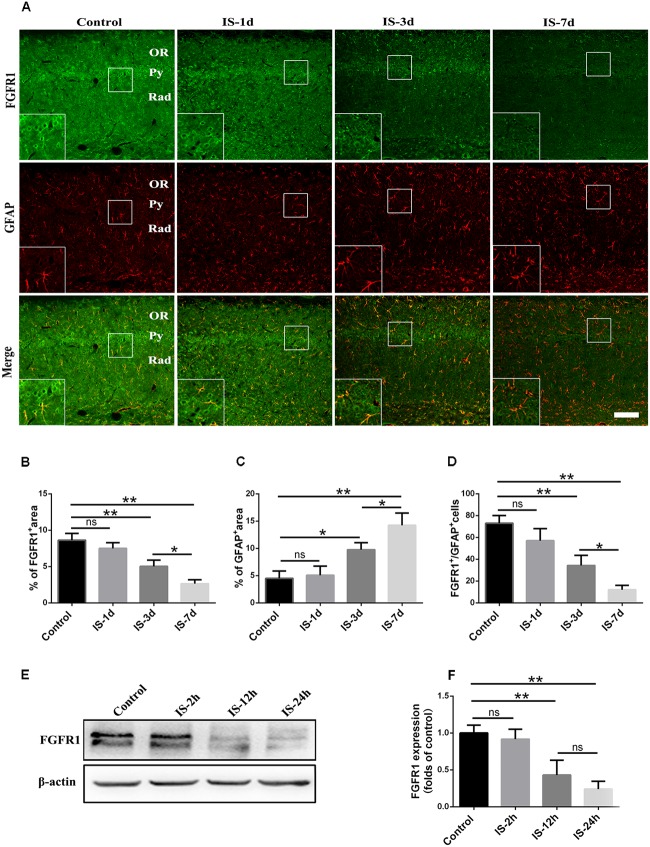
Changes in the expression of FGFR1 after infrasound exposure. **(A)** The changes in the expression of FGFR1 (green) and GFAP (red) revealed by double immunostaining around the CA1 region of rats at 1d (IS-1d), 3d (IS-3d), or 7d (IS-7d) post-exposure to infrasound. OR, oriens layer of the hippocampus; Py, pyramidal cell layer of the hippocampus; Rad, stratum radiatum of the hippocampus. Scale bar: 100 μm. **(B,C)** Quantitation of the percentage of FGFR1^+^ area and GFAP^+^ area around the hippocampus CA1 region (620 μm × 620 μm). **(D)** Count of the number of FGFR1^+^/GFAP^+^ cells around the hippocampal CA1 region (620 μm × 620 μm). **(E,F)** Western blotting revealed the changes in FGFR1 expression in primary cultured astrocytes at 2 h (IS-2h), 12 h (IS-12h), or 24 h (IS-24h) post-exposure to infrasound. β-Actin is as an internal control. All the data are represented as means ± SD. ^∗^*p* < 0.05, ^∗∗^*p* < 0.01. ns, no significance.

Consistent with our previous study (Shi et al., 2013), astrocytic activation was evidence, as indicated by increased GFAP expression (**Figures 1A,C**) after infrasound exposure. By contrast, the expression of FGFR1 were significantly decreased after 3 days, 7 days post-exposure to infrasound, as compared with control group (*p* < 0.01) (**Figures 1A,B**). Compared with the control group (73.00 ± 7.00), the number of FGFR1^+^/GFAP^+^ cells was also decreased at 7 days post-exposure to infrasound (12.00 ± 4.00, *p* < 0.01) (**Figures 1A,D**). Furthermore, in primary cultured astrocytes, the expression of FGFR1 and the number of FGFR1^+^/GFAP^+^ cells were also reduced at 12 h (2.75 ± 0.48, *p* < 0.05), 24 h (1.81 ± 0.38, *p* < 0.01) post-exposure to infrasound, compared with the control group (4.61 ± 0.79) (**Figures 1E,F** and **Supplementary Figure S2**). Therefore, these results suggested that after infrasound exposure, astrocyte-expressed FGFR1 was decreased *in vivo* and *in vitro*.

### FGF2 Attenuates Infrasound-Induced Astrocytic Activation

**FIGURE 2:**
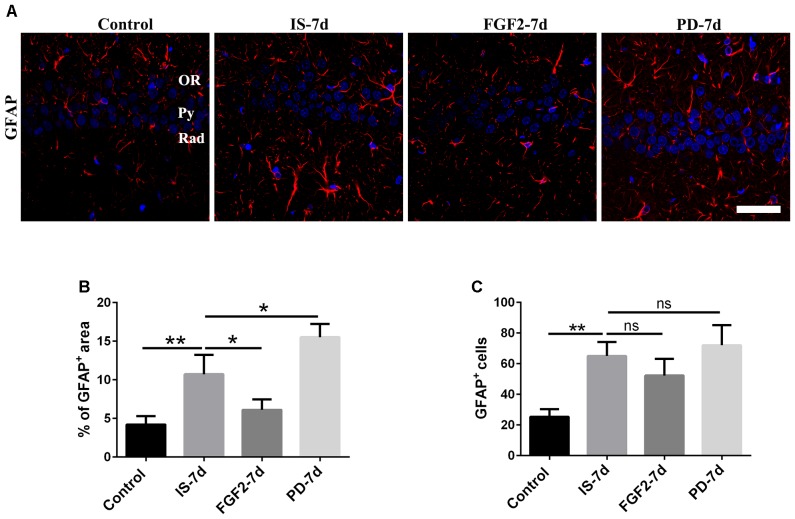
FGF2 attenuates astrocyte activation around the CA1 region of rats after infrasound exposure. **(A)** The changes in cells morphology revealed by GFAP (red) immunostaining around rat CA1 region with FGF2 (FGF2-7d) or PD173074 (PD-7d) treatment after 7 days of infrasound exposure (IS-7d). OR, oriens layer of the hippocampus; Py, pyramidal cell layer of the hippocampus; Rad, stratum radiatum of the hippocampus. Scale bar: 50 μm. **(B)** Quantitation of the percentage of GFAP^+^ area around the hippocampus CA1 region (620 μm × 620 μm). **(C)** Count of the number of FGFR1^+^/GFAP^+^ cells around the hippocampal CA1 region (620 μm × 620 μm). All the data are represented as means ± SD. ^∗^*p* < 0.05, ^∗∗^*p* < 0.01. ns, no significance.

To investigate the effects of FGF2 on infrasound-induced astrocyte activation, the rats were treated with exogenous FGF2 intraperitoneally. At post-7d exposure, the morphology of astrocytes around hippocampal CA1 region revealed by GFAP immunofluorescence was observed. We found that the processes of astrocytes significantly were longer and their bodies became larger than those of the control group. After pretreatment with FGF2, the processes of astrocytes became shorter and bodies became smaller (**Figure 2A**). Consistently, GFAP immunoreactivity and GFAP^+^ cells number were markedly increased after infrasound exposure (*p* < 0.01). FGF2 pretreatment attenuated infrasound-induced increased GFAP immunoreactivity (*p* < 0.05), and however, did not affect GFAP^+^ cells number (*p* > 0.05) (**Figures 2B,C**). We also have preformed the double-staining of GFAP and cleaved caspase-3 (a marker for apoptotic cells), and found no overt colocalization after FGF2 pretreatment (**Supplementary Figure S3**). Thus, we proposed that the reduction of GFAP^+^ area after FGF2 pretreatment did not result from the cells death but from the inhibition of astrocytes activation. Our results showed that infrasound exposure induced astrocyte activation and downregulated FGFR1 expression, and however, FGF2 administration could alleviate astrocyte activation, indicating that FGF2 may exert an inhibitory effect on infrasound-induced astrocyte activation.

### FGF2 Inhibits Infrasound-Triggered Elevation of Pro-inflammatory Cytokines

**FIGURE 3:**
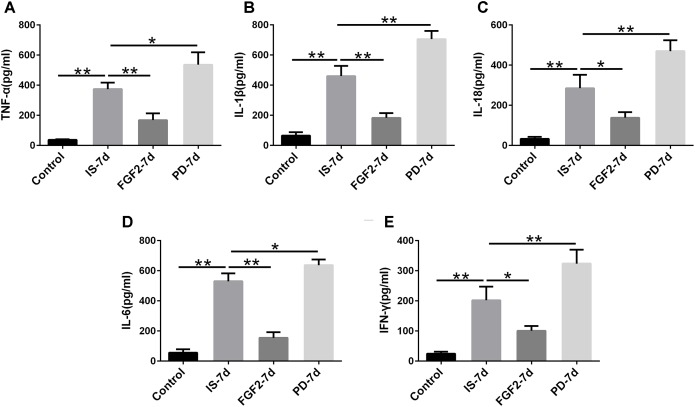
The levels of TNF-α, IL-1β, IL-18, IL-6, IFN-γ in the hippocampus of rats. Effect of FGF2 on infrasound-triggered pro-inflammatory cytokines release, including TNF-α **(A)**, IL-1β **(B)**, IL-18 **(C)**, IL-6 **(D)**, IFN-γ **(E)**. IS-7d means 7d post-infrasound exposure, FGF2-7d means FGF2 treated for 7d post-infrasound exposure, PD-7d means PD173074 treated for 7d post-infrasound exposure. All the data are represented as means ± SD. ^∗^*p* < 0.05, ^∗∗^*p* < 0.01.

Next, we examined whether FGF2 had an effect on glia-mediated inflammatory reaction, indicated by the levels of pro-inflammatory cytokines after infrasound exposure. Our results showed that the levels of TNF-α (*p* < 0.01), IL-1β (*p* < 0.01), IL-18 (*p* < 0.01), IL-6 (*p* < 0.01), and IFN-γ (*p* < 0.01) were significantly elevated in the hippocampus of rats after being exposed to infrasound at 7 days (**Figure 3**), compared with the control group. On the contrary, FGF2 administration inhibited the infrasound-mediated release of pro-inflammatory cytokines like TNF-α (*p* < 0.01), IL-1β (*p* < 0.01), IL-18 (*p* < 0.05), IL-6 (*p* < 0.01), and IFN-γ (*p* < 0.05), as compared with IS-7d group (**Figure 3**). To confirm these results, cytokines in the supernatant of infrasound-exposed astrocyte culture were examined. As expected, the levels of pro-inflammatory cytokines above were also remarkably increased after being exposed to infrasound at 12 h, compared with the control group (**Supplementary Figure S4**). However, cytokine levels were not statistically different between 12 and 24 h after infrasound exposure (data not shown). FGF2 pretreatment restrained the release of pro-inflammatory cytokines induced by infrasound (**Supplementary Figure S4**). Therefore, these results showed that FGF2 inhibited infrasound-triggered release of pro-inflammatory cytokines *in vivo* and *in vitro.*

### FGF2 Upregulates FGFR1 Expression After Infrasound Exposure

**FIGURE 4:**
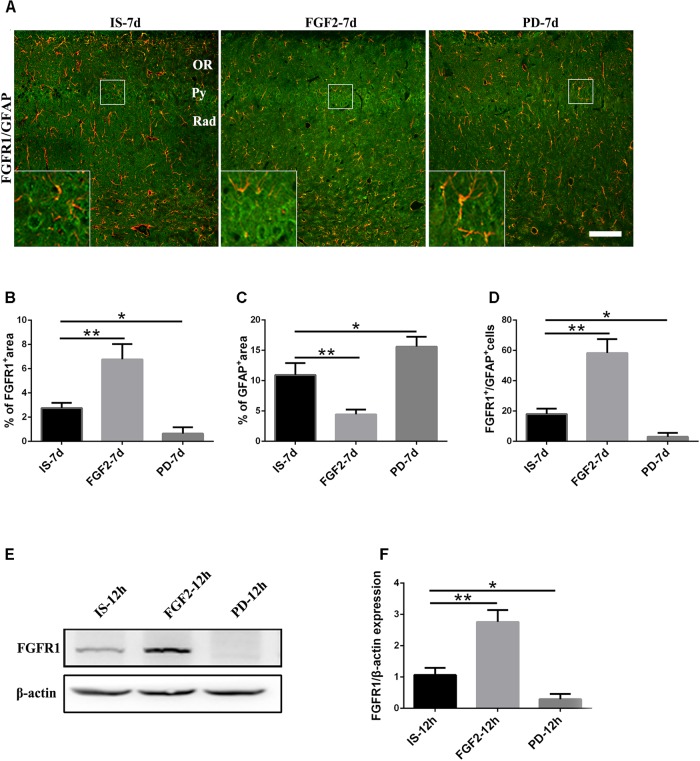
Changes in the expression of FGFR1 after FGF2 administration. **(A)** The changes in the expression of FGFR1 (green) and GFAP (red) revealed by double immunostaining around the CA1 region in rats with FGF2 (FGF2-7d) or PD173074 (PD-7d) treatment after 7d of infrasound exposure (IS-7d). OR, oriens layer of the hippocampus; Py, pyramidal cell layer of the hippocampus; Rad, stratum radiatum of the hippocampus. Scale bar: 100 μm. **(B)** Quantitation of the percentage of FGFR1^+^ area around the hippocampus CA1 region (620 μm × 620 μm). **(C)** Count of the number of FGFR1^+^/GFAP^+^ cells around the hippocampal CA1 region (620 μm × 620 μm). **(D,E)** Western blotting revealed the changes in FGFR1 expression in primary cultured astrocytes with FGF2 (FGF2-12h) or PD173074 (PD-12h) treatment after 12 h of infrasound exposure (IS-12h). β-Actin is as an internal control. All the data are represented as means ± SD. ^∗^*p* < 0.05, ^∗∗^*p* < 0.01.

Previous studies have showed that FGF2 could exert a positive feedback effect by upregulating FGFR1, its own receptor (Woodbury and Ikezu, 2014; Porta et al., 2017; Yuan et al., 2017). Therefore, we examined whether exogenous FGF2 also changed FGFR1 expression after infrasound exposure. As shown above, the expression of FGFR1 around the hippocampal CA1 region of rats was decreased to the lowest level at 7 days after infrasound exposure (*p* < 0.05), as compared with IS-3d group (**Figure 1A**). Therefore, we chose 7 days for the following study. FGF2 treatment increased FGFR1 immunoreactivity and FGFR1^+^/GFAP^+^ co-labeled cells around the CA1 region (**Figure 4A**). In addition, our *in vitro* results of Western blotting and immunofluorescence showed that after 12 h exposure to infrasound, FGFR1 expression in primary cultured astrocytes with FGF2 preconditioning was increased, as compared with that without FGF2 pretreatment (**Figure 4E** and **Supplementary Figure S5**). Taken together, our results showed that FGF2 remarkably upregulated FGFR1 expression both *in vivo* and *in vitro* after infrasound exposures.

### FGF2 Inhibits IκBα Phosphorylation and NF-κB Nuclear Translocation After Infrasound Exposure

As aforementioned, exogenous FGF2 could increase FGFR1 expression, and attenuate astrocyte-mediated inflammation after infrasound exposure, but the specific mechanism was still not clear. It has been reported that FGF2 could accelerate rat skin wound repair and regulate inflammatory response by inhibiting NF-κB p65 expression (Xuan et al., 2016). Thus, next we investigated whether FGF2/FGFR1 pathway could inhibit infrasound-triggered inflammation via NF-κB pathway as well.

**FIGURE 5:**
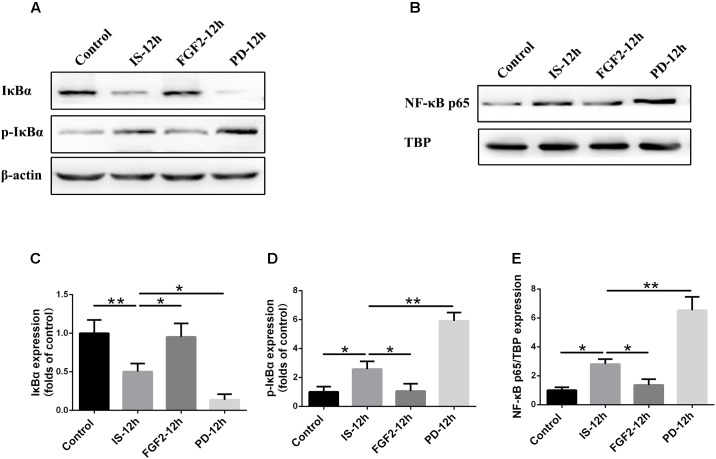
Effect of FGF2 administration on the expression of IκBα and NF-κB in the cultured astrocytes after infrasound exposure. **(A)** Western blotting revealed the changes in IκBα and p-IκBα expression in the cytoplasm of cultured astrocytes with FGF2 (FGF2-12h) or PD173074 (PD-12h) treatment after 12 h of infrasound exposure (IS-12h). **(B)** Western blotting revealed the changes in NF-κB p65 expression in the nucleus of cultured astrocytes with FGF2 (FGF2-12h) or PD173074 (PD-12h) treatment after 12h of infrasound exposure (IS-12h). **(C–E)** The changes in IκBα, p-IκBα, and NF-κB p65 expression in primary cultured astrocytes revealed at IS-12h group, PD-12h group and FGF2-12h group. β-Actin is as an internal control of cytoplasmic protein **(A)**. TBP is as an internal control of nuclear protein **(B)**. All the data are represented as means ± SD. Data are all expressed as means ± SD. ^∗^*p* < 0.05, ^∗∗^*p* < 0.01.

As shown above, FGFR1 expression in cultured astrocytes decreased strikingly after 12 h post-exposure to infrasound, as compared with the control group (*p* < 0.01) (**Figure 1E** and **Supplementary Figure S2**), and then we chose 12 h as the time point for the following study. Immunoblotting results showed that the level of IκBα phosphorylation and the expression of NF-κB p65 in the nuclei were increased at 12 h post-exposure to infrasound (*p* < 0.05), compared with the control group. On the contrary, after pretreatment of primary cultured astrocytes with FGF2, IκBα phosphorylation and nuclear p65 expression were obviously decreased (*p* < 0.05), compared with the infrasound group (**Figure 5**). Therefore, exogenous FGF2 may block infrasound-triggered NF-κB pathway.

### FGF2 Alleviates Neuron Loss in Hippocampal CA1 Region After Infrasound Exposure

**FIGURE 6:**
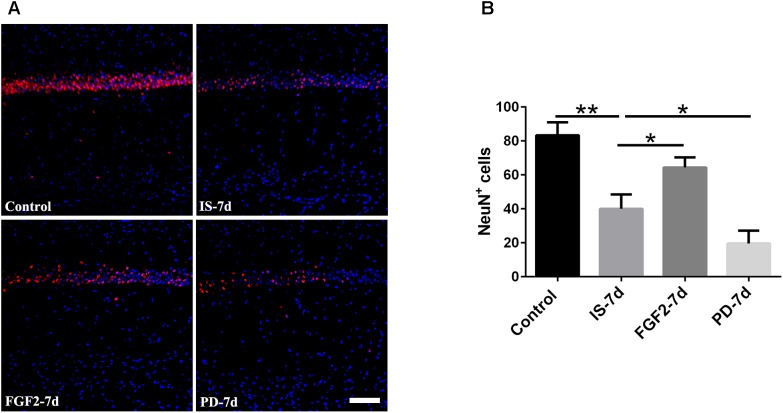
Changes in the number of NeuN^+^ cells after FGF2 administration. **(A)** The changes in the number of neuron revealed by NeuN (red) immunostaining in rat CA1 region with FGF2 (FGF2-7d) or PD173074 (PD-7d) treatment after 7d of infrasound exposure (IS-7d). Scale bar: 100 μm. **(B)** Count of the number of FGFR1^+^/GFAP^+^ cells in the hippocampal CA1 region (620 μm × 620 μm). All the data are represented as means ± SD. ^∗^*p* < 0.05, ^∗∗^*p* < 0.01.

Our previous study showed that inflammatory cytokines from infrasound exposure can cause neuron loss (Shi et al., 2013). Consistently, our present data also showed that the number of neurons indicated by NeuN immunostaining was obviously decreased in rat hippocampal CA1 region at 7 days post-exposure to infrasound, compared with the control group. As expected, double-labeling immunofluorescence of cleaved caspase-3 and NeuN showed that infrasound exposure increased the neuronal apoptosis (**Supplementary Figure S6**). After pretreatment with FGF2, the number of neurons (64.33 ± 6.03) was more than that exposed to infrasound (40.00 ± 8.54, *p* < 0.05) (**Figure 6**). Taken together, FGF2 could alleviate neuron loss in hippocampal CA1 region after infrasound exposure.

### FGF2/FGFR1 Pathway Inhibition Aggravates Infrasound-Induced Inflammation

In order to confirm the effects of FGF2/FGFR1 pathway after infrasound exposure, PD173074, a special antagonist of FGFR1, was administered. Our results showed that PD173074 aggravated infrasound-induced astrocyte activation and release of proinflammatory cytokines TNF-α, IL-1β, IL-18, IL-6, and IFN-γ *in vivo* and *in vitro* (**Figures 2**, **3** and **Supplementary Figure S4**). Moreover, PD173074 downregulated FGFR1 expression in astrocytes, compared with the infrasound group *in vivo* and *in vitro* (**Figure 4** and **Supplementary Figure S5**), and subsequently decreased IκBα phosphorylation (*p* < 0.01), increased nuclear expression level of NF-κB p65 (*p* < 0.01) (**Figure 5**). Moreover, precondition with PD173074 aggravated neuron loss, as compared with that exposed to infrasound (*p* < 0.05) (**Figure 6**). Taken together, inhibition of FGF2/FGFR1 pathway could aggravate astrocyte-mediated inflammation after infrasound exposure.

## Discussion

In this study, we investigated possible effects of FGF2/FGFR1 pathway in infrasound-induced inflammatory injury. Our results revealed that the exposure to16 Hz, 150 dB infrasound *in vivo* and *in vitro* could result in astrocytic activation and a decrease of FGFR1 expression. Activating FGF2/FGFR1 pathway by FGF2 attenuated astrocytes-mediated inflammation while FGFR1 inhibition aggravated these effects after infrasound exposure.

Astrocytes are the most widely distributed cells in the brain of mammals and are the key components in the brain inflammatory response (Bellaver et al., 2015; Colombo and Farina, 2016; Gonzalez-Reyes et al., 2017). Astrocytes are known to be divided into two major types, protoplasmic astrocytes in gray matter and fibrous astrocytes in white matter which are associated with the blood–brain barrier (Chen and Swanson, 2003; Freeman, 2010). Protoplasmic astrocytes which play a crucial role, can differentiate into fibrous astrocytes during brain ischemia injury (Kajihara et al., 2001; Lukaszevicz et al., 2002; Bylicky et al., 2018). Activation of astrocytes can promote the production of pro-inflammatory cytokines in pathological conditions (Sofroniew, 2015; Jha et al., 2017; Bylicky et al., 2018). In the present study, we demonstrated that after infrasound exposure, astrocyte activation was significant and the levels of TNF-α, IL-1β, IL-18, IL-6, and IFN-γ were significantly increased *in vivo* and *in vitro*, compared to the control group. These pro-inflammatory cytokines had turned out to have a harmful effect on neurons, leading to neuronal apoptosis (Lu et al., 2005; Cai et al., 2014; Phuagkhaopong et al., 2017; Yin et al., 2017), which was confirmed by our present results.

For exploration of possible mechanism underlying astrocytes-mediated inflammation after infrasound exposure, we focused on FGF2/FGFR1 pathway due to its important role in neuroinflammation. FGFR1, a tumorigenic receptor tyrosine kinase, plays an important role in some physiological processes and progression of cancer (Woodbury and Ikezu, 2014; Haenzi and Moon, 2017; Tang C. et al., 2017). In some conditions, FGFR1 signaling can be stimulated by FGF2, which can lead to dimerization and dephosphorylation of FGFR1, exhibiting corresponding actions, not only including cell proliferation, cell growth, anti-apoptosis, but also anti-inflammation (Yoshimura et al., 2001; Zittermann and Issekutz, 2006; Sun et al., 2017; Tang M.M. et al., 2017). In addition, FGF2/FGFR1 signaling could regulate inflammation of hippocampus to exert a positive effect on some neurodegenerative diseases such as Parkinson’s disease, Alzheimer’s disease, multiple sclerosis, and traumatic brain injury (Woodbury and Ikezu, 2014). In the present study, we showed that FGFR1 was expressed on astrocytes, and activated FGF2/FGFR1 signaling by FGF2 alleviated enhanced astrocytic activation and increased levels of TNF-α, IL-1β, IL-18, IL-6, IFN-γ induced by infrasound. Moreover, FGF2 could upregulate the expression of FGFR1 on astrocytes, implying that FGF2 maybe exert a positive feedback effect by upregulating its own receptor FGFR1 in infrasound-induced inflammation.

As for the mechanism underlying FGF2/FGFR1 signaling on anti-inflammation, NF-κB pathway may exert an important role (Xuan et al., 2016). NF-κB can regulate many processes involved in immune function and inflammation (Ho et al., 2012; Kim et al., 2017). Our results showed that after infrasound exposure, the expression of p-IκBα and nuclear NF-κB p65 was increased, suggesting that infrasound may activate NF-κB through degradation of IκBs and subsequent translocation of NF-κB p65 subunit into the nuclei, finally activating transcription of target genes, including various proinflammatory cytokines (such as TNF-α, IL-1β, IL-18, IL-6, and IFN-γ). After FGF2 administration, IκBα phosphorylation and p65 nuclear translocation were attenuated, the levels of pro-inflammatory cytokines were reduced, and the number of neuronal cells was increased, as compared with infrasound group. On the contrary, inhibition of FGF2/FGFR1 signaling by PD173074 reversed corresponding effects above. Thus, we proposed that FGF2 could attenuate the infrasound-induced inflammation by preventing the translocation of NF-κB into nucleus via the regulation of the IκBα.

Our results provided the first evidence that FGF2/FGFR1 pathway may exert an inhibitive effect on astrocyte-mediated inflammation *in vivo* and *in vitro* after infrasound exposure. Specifically, infrasound could downregulate FGFR1 expression and trigger astrocyte activation, NF-κB nuclear translocation, pro-inflammatory cytokine release and neuron loss. Activation of FGF2/FGFR1 pathway attenuated astrocytes-mediated inflammation while inhibition aggravated these effects induced by infrasound, suggesting that FGF2/FGFR1 pathway might be a promising target for treatment of infrasound-associated diseases.

## Conclusion

Our results provided the first evidence that astrocytes-mediated inflammation was involved in infrasound-induced neuronal damage, and FGF2/FGFR1 pathway may exert an inhibitive effect on this process.

## Ethics Statement

The experimental rules and regulations was approved by the Ethics Committee of the Affiliated Xijing Hospital of Fourth Military Medical University. The animal experiment for our study was approved by the Fourth Military Medical University Committee for Animal Research. All efforts were made to minimize animal suffering and to reduce the number of animals used.

## Author Contributions

LM and GZ conceived and designed the experiments. Y-JS performed the experiments. L-JX, LLi, and L-HZ analyzed the data. HH, C-YL, Q-JZ, and L-FZ contributed reagents, materials, and analysis tools. Y-JS and MS wrote the paper. All authors contributed to the final approval of the manuscript.

## Conflict of Interest Statement

The authors declare that the research was conducted in the absence of any commercial or financial relationships that could be construed as a potential conflict of interest.

## Supplementary Material

FIGURE S1 Co-localization of FGFR1 and GFAP around the CA1 region of hippocampus. FGFR1 (**A**, green) was expressed in astrocytes (**B**, red). Nucleus was represented by hoechst (**C**, blue). Co-localization of FGFR1^+^/GFAP^+^ **(D)** were shown as above. Scale bar: 10 μm.

FIGURE S2 Changes in the expression of FGFR1 in the primary cultured astrocytes after infrasound exposure. **(A)** The changes in the expression of FGFR1 (green) and GFAP (red) revealed by double immunostaining of FGFR1 with GFAP at control group, 2 h (IS-2 h), 12 h (IS-12 h), 24 h (IS-24 h) post-exposure to infrasound. IS-2 h means 2 h post-infrasound exposure and so on. Scale bar: 100 μm. **(B–D)** Quantitation of the percentage of FGFR1^+^ area, GFAP^+^ area and FGFR1^+^/GFAP^+^ area (620 μm × 620 μm) in the primary cultured astrocytes at different time points after infrasound exposure. All the data are represented as means ± SD. ^∗^*p* < 0.05, ^∗∗^*p* < 0.01. ns: no significance.

FIGURE S3 Double immunostaining of GFAP and cleaved caspase-3 around the CA1 region of hippocampus after FGF2 administration by infrasound exposure. GFAP (**A**, red) was not co-localizated with cleaved caspase-3 (**B**, green). Nucleus was represented by hoechst (**C**, blue). GFAP and cleaved caspase-3 (**D**) were not co-localizated. Scale bar: 10 μm.

FIGURE S4 The levels of TNF-α, IL-1β, IL-18, IL-6, IFN-γ in the primary cultured astrocytes. **(A–E)** Effect of FGF2 on infrasound-triggered pro-inflammatory cytokines release, including TNF-α **(A)**, IL-1β **(B)**, IL-18 **(C)**, IL-6 **(D)**, IFN-γ **(E)**. IS-12 h means 12 h post-infrasound exposure, FGF2-12 h means FGF2 treated for 12 h post-infrasound exposure, PD-12 h means PD173074 treated for 12 h post-infrasound exposure. All the data are represented as means ± SD. ^∗^*p* < 0.05, ^∗∗^*p* < 0.01.

FIGURE S5 Changes in the expression of FGFR1 after FGF2 administration by infrasound exposure. **(A)** The changes in the expression of FGFR1 (green) and GFAP (red) revealed by double immunostaining of FGFR1 with GFAP at 12 h post-exposure to infrasound (IS-12 h), FGF2 treated for 12 h post-infrasound exposure (FGF2-12 h), PD173074 treated for 12 h post-infrasound exposure (PD-12 h). Scale bar: 100 μm. **(B–D)** Quantitation of the percentage of FGFR1^+^ area, GFAP^+^ area and FGFR1^+^/GFAP^+^ area (620 μm × 620 μm) in the primary cultured astrocytes at different groups after infrasound exposure. All the data are represented as means ± SD. ^∗^*p* < 0.05, ^∗∗^*p* < 0.01.

FIGURE S6 Co-localization of NeuN (**A**, red) and cleaved caspase-3 (**B**, green) around the CA1 region of hippocampus after infrasound exposure. Nucleus was represented by hoechst (**C**, blue). Co-localization of NeuN^+^/cleaved caspase-3^+^ **(D)** were marked by arrows. Scale bar: 10 μm.
