# Supplementary figures and images for: Inhibitive Effects of FGF2/FGFR1 Pathway on Astrocyte-Mediated Inflammation in vivo and in vitro After Infrasound Exposure
Source: Front Neurosci. 2018 Aug 24;12:582. doi: 10.3389/fnins.2018.00582 (PMC6119807; doi:10.3389/fnins.2018.00582)

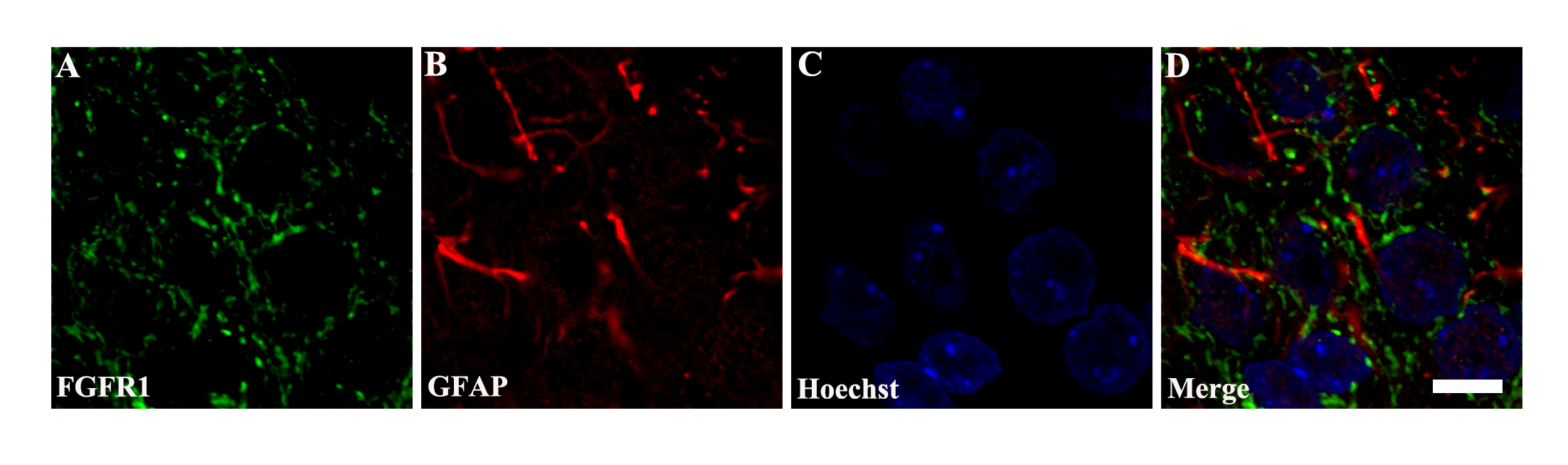

Supplement: FIGURE S1 — Co-localization of FGFR1 and GFAP around the CA1 region of hippocampus. FGFR1 (A, green) was expressed in astrocytes (B, red). Nucleus was represented by hoechst (C, blue). Co-localization of FGFR1+/GFAP+ (D) were shown as above. Scale bar: 10 μm. [file Image_1.TIF]

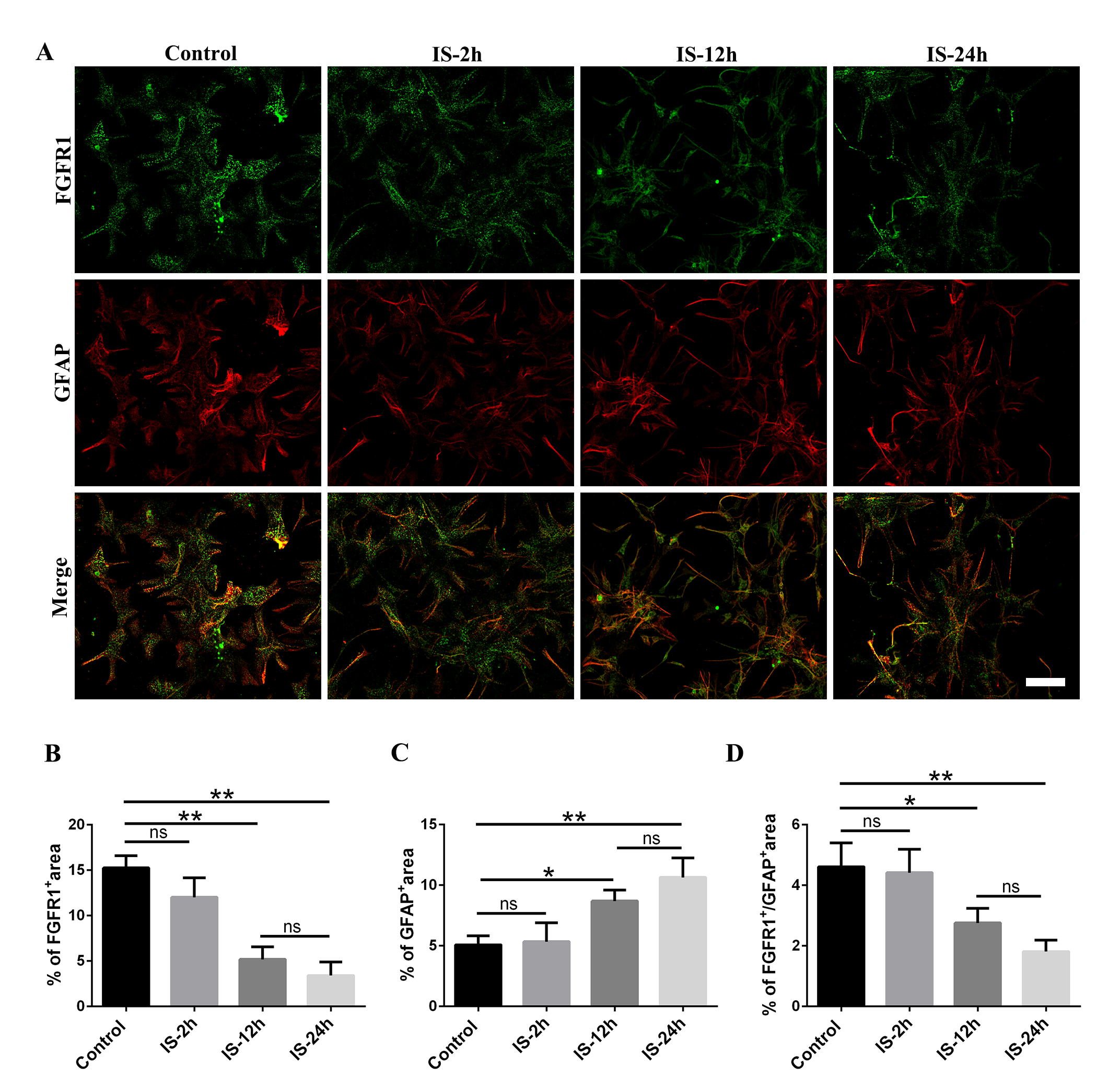

Supplement: FIGURE S2 — Changes in the expression of FGFR1 in the primary cultured astrocytes after infrasound exposure. (A) The changes in the expression of FGFR1 (green) and GFAP (red) revealed by double immunostaining of FGFR1 with GFAP at control group, 2 h (IS-2 h), 12 h (IS-12 h), 24 h (IS-24 h) post-exposure to infrasound. IS-2 h means 2 h post-infrasound exposure and so on. Scale bar: 100 μm. (B–D) Quantitation of the percentage of FGFR1+ area, GFAP+ area and FGFR1+/GFAP+ area (620 μm × 620 μm) in the primary cultured astrocytes at different time points after infrasound exposure. All the data are represented as means ± SD. ∗p < 0.05, ∗∗p < 0.01. ns: no significance. [file Image_2.TIF]

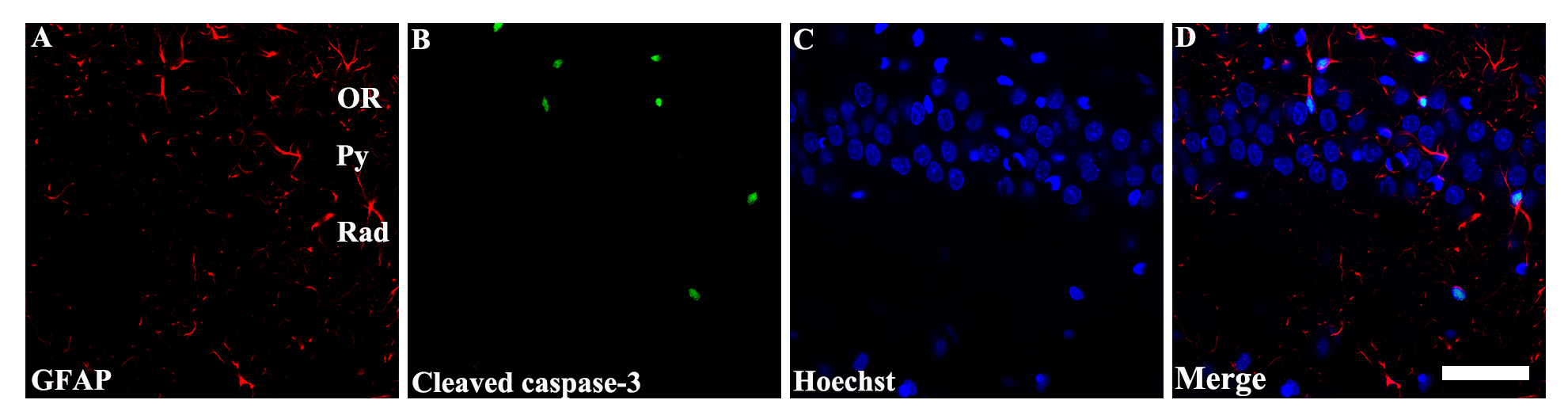

Supplement: FIGURE S3 — Double immunostaining of GFAP and cleaved caspase-3 around the CA1 region of hippocampus after FGF2 administration by infrasound exposure. GFAP (A, red) was not co-localizated with cleaved caspase-3 (B, green). Nucleus was represented by hoechst (C, blue). GFAP and cleaved caspase-3 (D) were not co-localizated. Scale bar: 10 μm. [file Image_3.TIF]

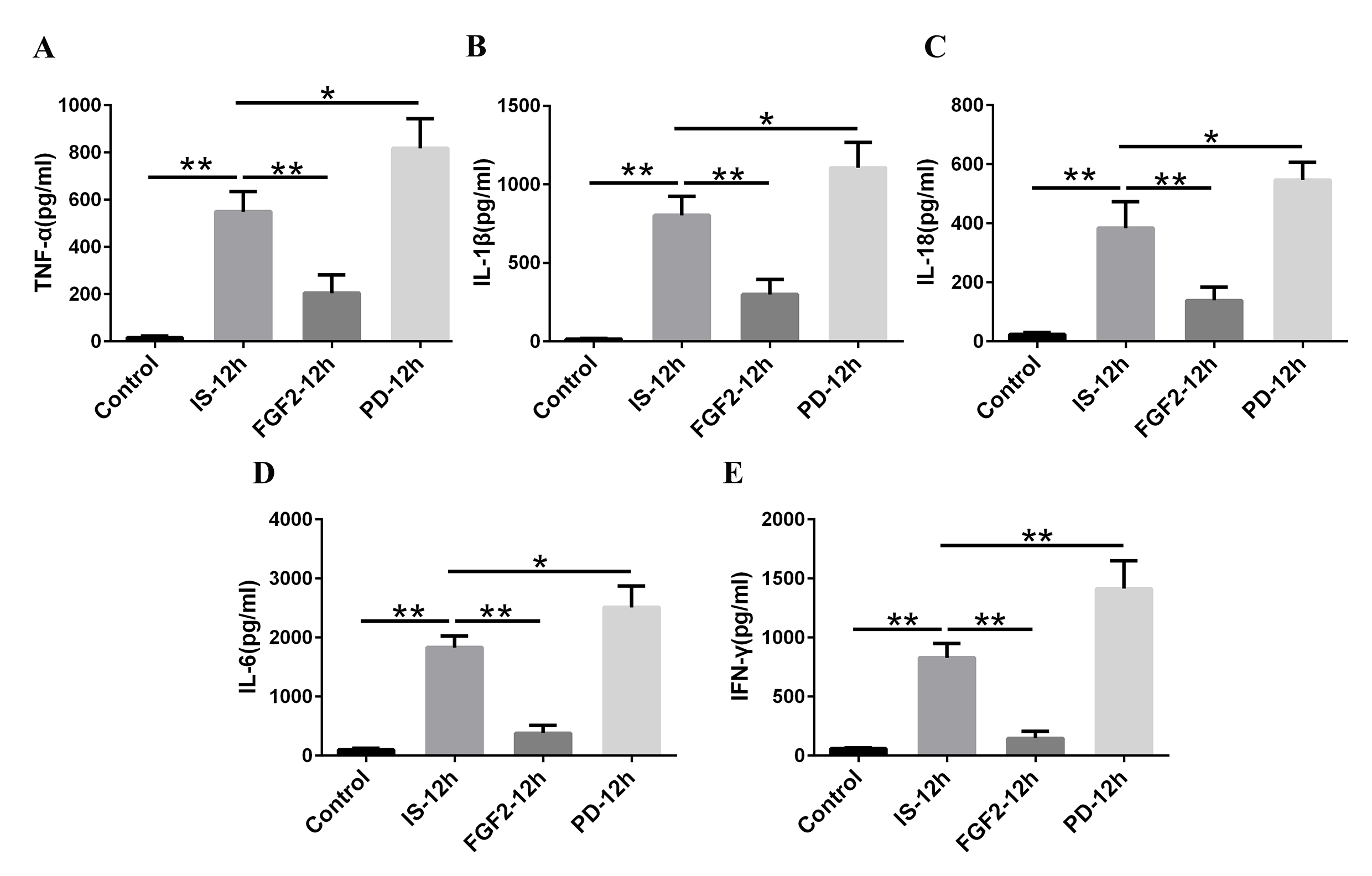

Supplement: FIGURE S4 — The levels of TNF-α, IL-1β, IL-18, IL-6, IFN-γ in the primary cultured astrocytes. (A–E) Effect of FGF2 on infrasound-triggered pro-inflammatory cytokines release, including TNF-α (A), IL-1β (B), IL-18 (C), IL-6 (D), IFN-γ (E). IS-12 h means 12 h post-infrasound exposure, FGF2-12 h means FGF2 treated for 12 h post-infrasound exposure, PD-12 h means PD173074 treated for 12 h post-infrasound exposure. All the data are represented as means ± SD. ∗p < 0.05, ∗∗p < 0.01. [file Image_4.TIF]

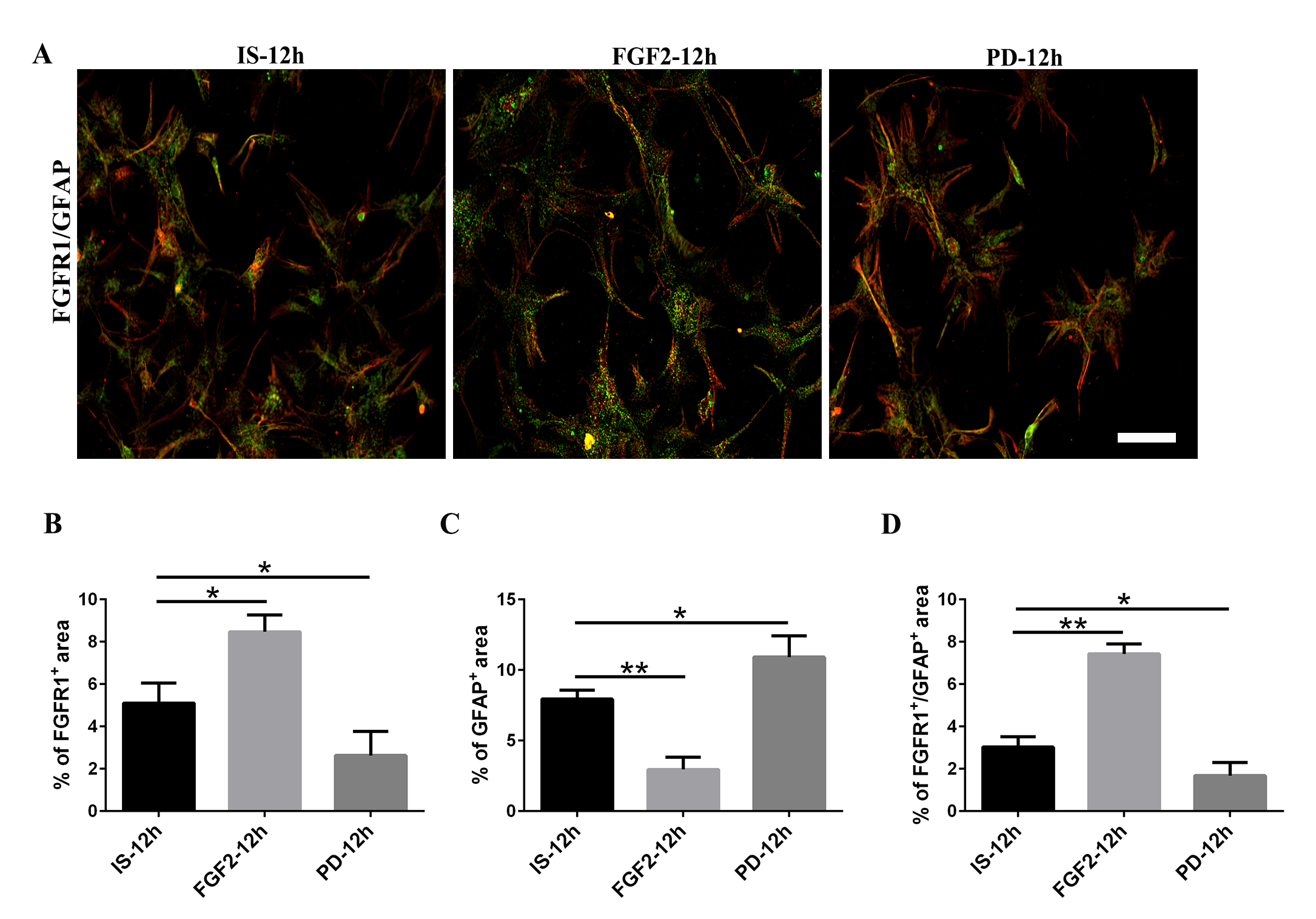

Supplement: FIGURE S5 — Changes in the expression of FGFR1 after FGF2 administration by infrasound exposure. (A) The changes in the expression of FGFR1 (green) and GFAP (red) revealed by double immunostaining of FGFR1 with GFAP at 12 h post-exposure to infrasound (IS-12 h), FGF2 treated for 12 h post-infrasound exposure (FGF2-12 h), PD173074 treated for 12 h post-infrasound exposure (PD-12 h). Scale bar: 100 μm. (B–D) Quantitation of the percentage of FGFR1+ area, GFAP+ area and FGFR1+/GFAP+ area (620 μm × 620 μm) in the primary cultured astrocytes at different groups after infrasound exposure. All the data are represented as means ± SD. ∗p < 0.05, ∗∗p < 0.01. [file Image_5.TIF]

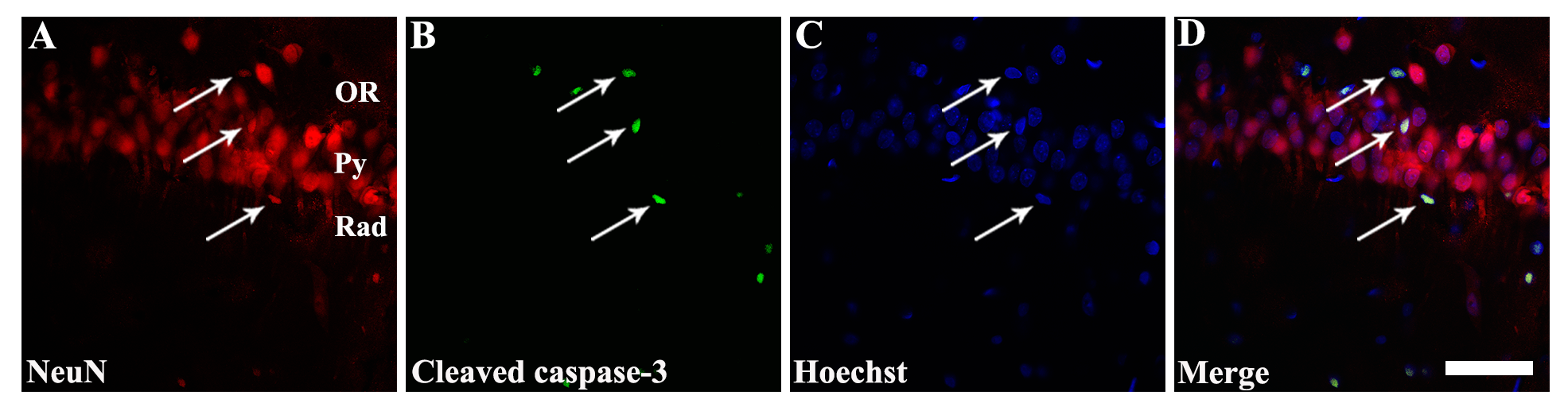

Supplement: FIGURE S6 — Co-localization of NeuN (A, red) and cleaved caspase-3 (B, green) around the CA1 region of hippocampus after infrasound exposure. Nucleus was represented by hoechst (C, blue). Co-localization of NeuN+/cleaved caspase-3+ (D) were marked by arrows. Scale bar: 10 μm. [file Image_6.TIF]
